# Polyglutamine Expansion in Huntingtin and Mechanism of DNA Damage Repair Defects in Huntington’s Disease

**DOI:** 10.3389/fncel.2022.837576

**Published:** 2022-04-04

**Authors:** Subrata Pradhan, Rui Gao, Keegan Bush, Nan Zhang, Yogesh P. Wairkar, Partha S. Sarkar

**Affiliations:** ^1^Department of Neurology, University of Texas Medical Branch, Galveston, TX, United States; ^2^Department of Neuroscience, Cell Biology and Anatomy, University of Texas Medical Branch, Galveston, TX, United States; ^3^Department of Neurology, Houston Methodist Research Institute, Houston, TX, United States

**Keywords:** defective DNA damage repair, huntingtin, Huntington’s disease, polyglutamine repeat expansion, transcription-coupled DNA repair, microsatellite repeat expansion

## Abstract

Emerging evidence suggests that DNA repair deficiency and genome instability may be the impending signs of many neurological diseases. Genome-wide association (GWAS) studies have established a strong correlation between genes that play a role in DNA damage repair and many neurodegenerative diseases, including Huntington’s disease (HD), and several other trinucleotides repeat expansion-related hereditary ataxias. Recently, many reports have documented a significant role played by the DNA repair processes in aging and in modifying many neurodegenerative diseases, early during their progression. Studies from our lab and others have now begun to understand the mechanisms that cause defective DNA repair in HD and surprisingly, many proteins that have a strong link to known neurodegenerative diseases seem to be important players in these cellular pathways. Mutations in *huntingtin* (*HTT*) gene that lead to polyglutamine repeat expansion at the N-terminal of HTT protein has been shown to disrupt transcription-coupled DNA repair process, a specialized DNA repair process associated with transcription. Due to the recent progress made in understanding the mechanisms of DNA repair in relation to HD, in this review, we will mainly focus on the mechanisms by which the wild-type huntingtin (HTT) protein helps in DNA repair during transcription, and the how polyglutamine expansions in HTT impedes this process in HD. Further studies that identify new players in DNA repair will help in our understanding of this process in neurons. Furthermore, it should help us understand how various DNA repair mechanism(s) coordinate to maintain the normal physiology of neurons, and provide insights for the development of novel drugs at prodromal stages of these neurodegenerative diseases.

## Introduction

Understanding of most neurodegenerative disorders suffer from the lack of the knowledge of their etiology. This creates a huge and significant challenge for designing and targeting effective therapies for the patients suffering from these untreatable, and terminal conditions. For example, although pathological features of Alzheimer’s disease (AD) are the deposition of Aβ peptides and tau aggregates, the therapies that have been directed toward these proteins have not had a remarkable success. Part of the problem could be that we are not targeting early etiological causes and missing opportunities to dramatically improve the patient outcomes by not targeting early causes of the disease. Recent studies have shown that substantial DNA damage occurs early in many neurodegenerative diseases (Rolig and McKinnon, 2000; McKinnon and Caldecott, 2007; McKinnon, 2009; Madabhushi et al., 2014), including AD (Shackelford, 2006; Shanbhag et al., 2019), Parkinson’s disease (PD; Milanese et al., 2018; Schaser et al., 2019; Martín-Jiménez et al., 2020; Gonzalez-Hunt and Sanders, 2021), Huntington’s disease (HD; Browne et al., 1997; Bogdanov et al., 2001; Giuliano et al., 2003; Illuzzi et al., 2009; Enokido et al., 2010; Bertoni et al., 2011; Tamura et al., 2011; Askeland et al., 2018; Massey and Jones, 2018), spinocerebellar ataxia type 3 (SCA3; Chatterjee et al., 2015; Gao et al., 2015; Chakraborty et al., 2020), and amyotrophic lateral sclerosis (ALS; Konopka and Atkin, 2018; Mitra et al., 2019; Konopka et al., 2020); while it is becoming increasingly clear that DNA damage may occur early and maybe an important early contributor to these diseases, it is unclear how DNA repair processes contribute to the dysfunction of neurons. Because our lab along with others, have made significant contributions toward understanding transcription-coupled DNA repair (TCR) processes in HD and other polyglutamine (polyQ) diseases, for the sake of this review, we will largely focus on DNA repair mechanisms and their relationship with TCR in HD.

Huntington’s disease is a rare genetic disorder with a prevalence of 5–10 individuals per 100,000 in the Caucasian population and many more people are at risk of developing the disease (Morrison, 2012; Baig et al., 2016; Rawlins et al., 2016). Some juvenile forms of the disease also exist but are rare, accounting for about 5% of the cases (Nance and Myers, 2001). The term “Juvenile HD” is applied to the cases of HD with disease onset before the age of 20. While HD symptoms that appear in adults primarily manifest with pure movement disorder or chorea (No authors listed, 1993; Vonsattel and DiFiglia, 1998; Rosenblatt et al., 2003; Ross and Tabrizi, 2011), patients with Juvenile HD develop symptoms of mental disturbance and rigidity rather than chorea (Nance and Myers, 2001; Geevasinga et al., 2006).

Huntington’s disease is a complex disease, and the disease symptoms vary between individuals but is typically manifested with three distinct sets of symptoms known as classical triad consisting of: (1) involuntary choreatic movements and motor coordination defects, (2) mild to moderate cognitive decline, and (3) psychiatric and behavioral abnormalities. At the onset, all the symptoms may not appear simultaneously, and symptoms often vary between individuals and the affected members of a family, however, the symptoms progress predictably with age (Ross et al., 1997; Walker, 2007). Typically, the phenotypes manifest in the middle-age (Ross et al., 1997; Walker, 2007) when they present with psychiatric and cognitive symptoms, although there are cases of juvenile onset at ages as young as 2 years (Nance and Myers, 2001). The motor coordination defects in adult-onset HD can be divided into the choreiform movements with gait disturbances that usually appear at the early stages of disease progression, and motor impairments such as bradykinesia and rigidity that are usually observed in the later stages of the disease (No authors listed, 1993; Vonsattel and DiFiglia, 1998; Rosenblatt et al., 2003; Ross and Tabrizi, 2011). Importantly, there is mounting evidence that cognitive deficits become apparent in mutant carriers several years prior to the onset of clinical symptoms (Jason et al., 1988; Hahn-Barma et al., 1998; Lawrence et al., 1998; Marder et al., 2000; Berrios et al., 2002). Cognitive impairment starts with subtle disturbances but progressively leads to detectable cognitive decline. In addition, HD patients cannot organize or plan simple tasks in daily life and lose flexibility of mind and often fail to make simple adjustments. Lastly, the HD patients can also be diagnosed with frequent depression, excessive mood swings and anxiety, and signs of apathy, irritability, impulsivity, and social disinhibition. Thus, HD is a complex disease, but subtle molecular changes likely occur well before the clinical manifestation of the disease, providing a window of opportunity for therapeutic interventions.

### Neurobiology of Huntington’s Disease

In a seminal discovery, the unique pathological mutation in HD was discovered in 1993 (No authors listed, 1993). This ground-breaking discovery was not only important in identifying a unique genetic mutation that causes human genetic disease and in uncovering the complex HD pathogenic mechanism(s) but also led the way in unraveling the underlying pathomechanism of several other polyglutamine (polyQ)-expansion related hereditary ataxias. The mutation associated with HD was found to be an expansion of a polyQ tract at the N-terminal coding region of the huntingtin (HTT) protein due to an expansion of a polymorphic CAG trinucleotide repeat sequences in the mutant huntingtin (*mHTT*) gene, which leads to progressive deterioration of cognitive and motor functions in patients with HD (No authors listed, 1993; Vonsattel and DiFiglia, 1998; Ross and Tabrizi, 2011). In normal population, the polymorphic CAG repeat sequences are in the range of 5–35 repeats, with a median length of 17–20 CAG repeats. Full penetrance is observed for mHTT alleles containing more than 40 CAG repeats (No authors listed, 1993). CAG repeat length of 36 or more at the N-terminal of HTT gene was found to be associated with adult-onset HD (No authors listed, 1993). An inverse correlation was observed between the length of the CAG repeats and the age of onset, determined by the first motor manifestation (No authors listed, 1993; Rosenblatt et al., 2006). In another interesting report, the length of uninterrupted CAG repeats in DNA, rather than the polyQ length at the N-terminal of HTT was found to be a critical contributing factor in HD disease onset (Genetic Modifiers of Huntington’s Disease [GeM-HD] Consortium, 2019).

### The Proposed Pathogenic Mechanism(s) of Huntington’s Disease

Clinical studies and studies in animal models of HD support the idea that mHTT protein carrying the extended stretch (more than 35 repeats) of polyQ sequences at the N-terminal region results in progressive degeneration of the gamma-aminobutyric acid (GABA)-releasing striatal neurons and glutamatergic cortical neurons in the basal ganglia. Additionally, neuronal dysfunction and tissue atrophy in other brain regions has also been reported in HD (Vonsattel and DiFiglia, 1998; Ross and Tabrizi, 2011).

**FIGURE 1:**
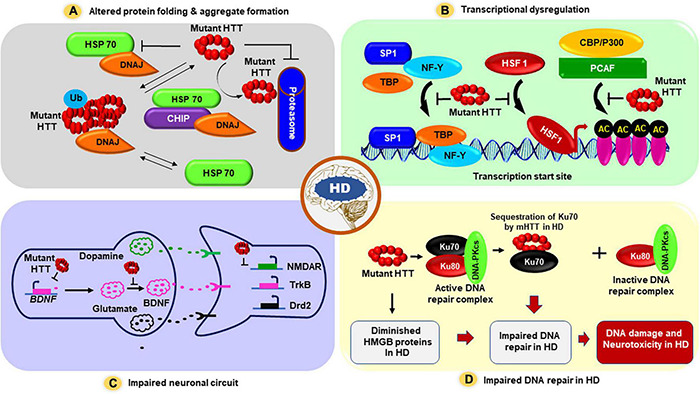
The proposed mechanism(s) by which mHTT triggers neuronal dysfunction and neurotoxicity in HD. Several possible pathogenic mechanism(s) for HD have been proposed: **(A)** First, mHTT can adopt altered structural conformations, and these inappropriately folded structures may aberrantly interact with many neuronal proteins, and these unusual protein-protein interactions may result in protein aggregate formation, and these aggregates may physically interfere with the vesicle and organelle transport within the cells, triggering neurotoxicity in HD. These aggregates may also fully or partially inactivate numerous proteins that are sequestered into HTT aggregates in HD neurons, contributing to neurotoxicity and HD pathogenesis. **(B)** mHTT carrying expanded polyQ sequences cause transcriptional dysregulation in HD: The mHTT protein translocate into the nucleus, interacts with various transcription factors and cofactors, and interferes with their normal functions, resulting in aberrant transcription of many neuronal genes that regulate neuronal function, survival, synaptic function, and vesicle transport. mHTT-mediated transcriptional dysregulation ultimately results in neuronal dysfunction, neurotoxicity, and neurodegeneration at early stages of disease progression in HD; **(C)** Evidence also suggests that in addition to overt transcription dysregulation, mHTT may also impair neuronal circuits and interfere with neurotransmission and synaptic function causing early neurotoxicity in HD. Hypothesized model illustrating how changes to the cortical-striatal synapse during early-stages of HD. In response to mHTT expression expression of BDNF is reduced in HD. Glial glutamate uptake by glutamate transporter 1 (GLT-1) is reduced, which probably enhances synaptic and/or extrasynaptic glutamate levels. Postsynaptic N-methyl-D-aspartate receptors (NMDAR) are increased outside the synapses, and gamma-aminobutyric acid (GABA)-argic inhibitory input is enhanced and tyrosine kinase B (TrkB) signaling is reduced. **(D)** Evidence also suggests that mHTT aberrantly interacts with and sequesters Ku70, an essential factor in NHEJ-mediated DNA double-strand break repair in HD. Sequestration of Ku70 by mHTT results in impaired DNA double-strand break repair in HD. Moreover, mHTT can also repress the expression of high mobility group proteins B1 and B2 (HMGB1/2), and mHTT-mediated downregulation of HMGB1/2 proteins can also result in defective DNA repair and DNA damage accumulation in HD. Abbreviations: SP1, Specificity protein 1; TBP, TATA binding protein; NF-Y, Nuclear factor Y; HSF1, Heat shock transcription factor 1; CBP, CREB-binding protein; AC, Acetylation; HSP70, Heat shock protein 70; DNAJ, Chaperone protein; Ub, Ubiquitination; BDNF, Brain-derived neurotrophic factor; NMDAR, *N*-methyl-D-aspartate receptors; TrkB, Tropomyosin receptor kinase B; Drd2, Dopamine Receptor D2.

Microscopic and molecular analyses of postmortem HD patient brains reveal the presence of aggregated form of mHTT (Davies et al., 1997; DiFiglia et al., 1997; Hoffner et al., 2007; Bauer and Nukina, 2009; Legleiter et al., 2010; Sontag et al., 2012). Based on these observations, it was hypothesized that the mHTT protein carrying extended stretch of polyQ sequences adopts unusual structural conformations, which facilitate aberrant protein–protein interactions. These result in the formation of large insoluble protein aggregates in the HD neurons. How do the aggregates affect the neuronal health? Many possibilities exist. First, the protein aggregates could negatively impact transcription of several neuronal genes because the aggregated HTT can interact and sequester key transcription factors and co-activators that regulate transcription of these genes (Luthi-Carter and Cha, 2003; Cha, 2007; Ross and Tabrizi, 2011; Kumar et al., 2014). Second, protein aggregates could physically interfere with the trafficking of organelles (Chang et al., 2006; Orr et al., 2008), and vesicles (Truant et al., 2006; Caviston and Holzbaur, 2009) in the affected neurons and glial cells (Figure 1). Third, HTT aggregates can interact with important signaling pathways (Davies et al., 1997; DiFiglia et al., 1997; Hoffner et al., 2007), can cause mitochondrial dysfunction and energy dyshomeostasis (Orr et al., 2008; Shirendeb et al., 2011; Siddiqui et al., 2012), and directly impact synaptic function (Morton et al., 2001; Li J. Y. et al., 2003; Joshi et al., 2009; Raymond et al., 2011). Finally, the aggregates could also lead to a disruption in DNA damage repair mechanism(s) leading to the excessive accumulation of DNA damage/strand breaks, leading to the chronic activation of the ataxia telangiectasia-mutated (ATM) and p53 signaling pathways (Bogdanov et al., 2001; Giuliano et al., 2003; Bae et al., 2005; Illuzzi et al., 2009; Bertoni et al., 2011; Lu et al., 2014; Askeland et al., 2018). Of interest, a recent study highlighting the importance of ATM pathway has demonstrated that either genetic or pharmacological ablation of ATM kinase activity significantly reduces the neurotoxicity in HD animal models (Lu et al., 2014). This study supports the emerging theme that persistent accumulation of DNA damage in neuronal DNA, and subsequent chronic activation of DNA damage-response (DDR) ATM pathway might be one of the important contributing factors to early pathogenesis of HD. However, whether accumulation of DNA damage is due to defective or impaired DNA repair mechanism(s), or chronic oxidative stress that stem from HTT protein aggregates remains a debatable.

In this review, we will briefly discuss some of the major pathogenic mechanisms that have been proposed for HD, with the focus on how HTT helps in DNA repair and genome maintenance. We will also comment upon the emerging view of and how mutant HTT (mHTT) protein impairs TCR, a specialized DNA repair mechanism in non-dividing postmitotic cells like neurons.

## Mutant Huntingtin Adopts Abnormal Conformation(s), and Is Deposited as Insoluble Aggregates in Huntington’s Disease Neurons

While the toxicity arising from the aggregates adopted by mHTT is largely hypothesized to be the cause and progression of the disease (Davies et al., 1997; DiFiglia et al., 1997; Benn et al., 2005; Hoffner et al., 2007; Sawada et al., 2007; Bauer and Nukina, 2009; Legleiter et al., 2010; Sontag et al., 2012), it is still a subject of intense debate whether the aggregates (Legleiter et al., 2010; Sontag et al., 2012) play any toxic role in HD pathogenic mechanisms or whether they are actually neuro protective. It also remains to be established whether the aggregates or oligomeric forms of the mutant protein are toxic for the HD neurons (Hoffner et al., 2007; Shirendeb et al., 2011). Arguments in favor of the aggregate hypothesis include interactions of HTT aggregates with various chaperone proteins, including heat shock proteins HSP40 and HSP70, and components of the neuronal proteasome system, resulting in sequestration of the proteins involved in protein clearance system. This might affect the protein folding machinery as well as clearance of the misfolded proteins in HD (Davies et al., 1997; DiFiglia et al., 1997; Muchowski et al., 2000). In fact, the HDJ-2 and HSP70 chaperones were found to co-localize with HTT aggregates in the brain of R6/2 HD transgenic mice expressing the N-terminal truncated fragment of mHTT (Mangiarini et al., 1996), and exogenous expression of chaperons HDJ-1 and HSP70 reduced the amount aggregates formed by mHTT and ameliorated the toxicity in cell models of HD (Jana et al., 2000). This unusual protein interactions might result in the inactivation of other key neuronal proteins adding to the complexity of the disease pathology. Importantly, increasing the levels of chaperons facilitates the clearance of the aggregates and reduces the neurotoxicity in HD models (Figure 1), suggesting that protein folding machinery and/or protein clearance systems are compromised in HD. However, the precise mechanisms of how mHTT disrupts these fundamental cellular processes in HD remains to be established.

## Mutant Huntingtin Interferes With the Transcription Process by Interacting With Many Transcription Factors and Cofactors in Huntington’s Disease

Over the last two decades it has become clear that both HTT and mHTT are present in the nucleus, directly interact with various transcription factors, cofactors, and RNA polymerase, and regulate transcription of neuronal genes (Cha, 2007; Benn et al., 2008; Ross and Tabrizi, 2011; Valor, 2015; Jimenez-Sanchez et al., 2017). Both HTT and mHTT interact with several nuclear transcription factors and cofactors, including cyclic AMP-response element-binding protein (CBP; McCampbell et al., 2000; Steffan et al., 2000; Nucifora et al., 2001), TATA-binding protein (TBP; Huang et al., 1998), the tumor suppressor transcription factor p53 (Steffan et al., 2000; Bae et al., 2005), the general transcription factors TFIID and TFIIF (Zhai et al., 2005), and specificity protein 1 (SP1; Dunah et al., 2002). The RNA polymerase large subunit A (POLR2A) also interacts with HTT and is detected in nuclear inclusions in the HD brain (Huang et al., 1998; Suhr et al., 2001). Additionally, the transcription cofactor TAF_*II*_130 (a cofactor for CREB-dependent transcription) has also been shown to bind to expanded polyQ sequences to interfere with CREB-dependent transcription in HD (Shimohata et al., 2000a). Moreover, peroxisome proliferator-activated receptor γ (PPARγ) coactivator 1α (PGC-1α), a transcriptional coactivator (Lin et al., 2002, 2005) that works in combination with other transcription factors like PPARγ may play an important role in HD pathogenesis (Cui et al., 2006; Weydt et al., 2006). mHTT has been shown to interfere with the binding of the CREB/TAF4 complex on PGC-1α promoter, repressing its expression, and evidence suggest that reduced PGC-1α level/activity might contribute to neurotoxicity in HD (Cui et al., 2006; Weydt et al., 2006). Moreover, the repressor element 1 (RE1)-silencing transcription (REST) is a master regulator of neuronal gene transcription, repressing their transcription (Huang et al., 1999). Several REST target genes e.g., BDNF (brain-derived neurotrophic factor) are known to be downregulated in HD, and reduced BDNF activity contribute to HD pathogenesis (Zuccato et al., 2001; Canals et al., 2004). Overexpression of BDNF in HD transgenic mouse brain can rescue HD-like phenotypes (Xie et al., 2010). Evidence also suggest that REST regulates transcription of microRNAs, many of which regulate neuronal gene expression and are dysregulated in HD (Johnson et al., 2008; Johnson and Buckley, 2009). Further, HTT has been shown to bind REST in the cytoplasm and thus prevents REST-mediated suppression of BDNF (Zuccato et al., 2003, 2007). The presence of mHTT results in reduced interaction between mHTT and REST, and consequently nuclear levels of REST increases, repressing BDNF expression (Zuccato et al., 2003, 2007). Based on these observations, it is hypothesized that HTT probably assists in the assembly of transcription factor and co-activator into multi-protein active transcription complexes, which regulate target gene expression in neurons, and that mHTT perturbs either the assembly or the function of these transcription complexes (Luthi-Carter and Cha, 2003; Ross and Tabrizi, 2011; Kumar et al., 2014; Figure 1).

## Mutant Huntingtin Causes Early Synaptic Dysfunction in Huntington’s Disease

There is ample evidence to suggest that subtle alterations of synapses occur before overt neuronal death, and that these changes are predictive of the onset of behavioral problems associated with HD (Morton et al., 2001; Li H. et al., 2003; Joshi et al., 2009; Raymond et al., 2011). Huntingtin is highly expressed in the presynaptic terminals of nerve cells (DiFiglia et al., 1995), and various proteins that regulate both the presynaptic and postsynaptic function have been shown to interact with HTT (Li et al., 2002; Li J. Y. et al., 2003; Barron et al., 2021). These proteins affect a range of pre and postsynaptic functions, including endocytosis, synaptic homeostasis, and axonal transport on the presynaptic side, and postsynaptic receptor localization and dendritic protein transport at the post-synapse.

At the presynaptic terminal, HTT can regulate the normal exocytosis, endocytosis, and axonal transport. For example, Complexin II – a protein involved in neurotransmitter release (Rizo and Südhof, 2002), is decreased in HD patients (Morton and Edwardson, 2001), R6/2 transgenic mice (Morton et al., 2001) and neuronal cell model of HD (Edwardson et al., 2003). Another protein involved in docking of synaptic vesicles to the membrane is rabphilin 3A, the level of which is progressively decreased in R6/1 mouse model of HD (Smith et al., 2005). Also, reduction of HTT-interacting proteins involved in endocytosis might impair endocytic and intracellular trafficking in HD (Kalchman et al., 1997; Li et al., 1998; Sittler et al., 1998; Modregger et al., 2002; Singaraja et al., 2002; Trushina et al., 2004). HTT-interacting protein 1 (HIP1) is closely related HIP12, and these proteins are orthologs of the yeast Sla2p, which is involved in endocytosis (Engqvist-Goldstein et al., 1999). HIP1 interacts with clathrin and AP-2, whereas HIP12 interacts with F-actin and clathrin light chain (Legendre-Guillemin et al., 2002). The most studied of these proteins is HTT-associated protein 1 (HAP1) (Li et al., 1995) that interacts with the p150 Glued subunit of dynactin (Engelender et al., 1997; Li et al., 1998), which in turn, interacts with dynein, the motor protein involved in retrograde transport. These two proteins interact to facilitate efficient vesicular transport along the microtubules (Gill et al., 1991). Studies have shown that the binding of HAP1 with HTT is increased in HD (Li et al., 1995), and increased binding of mHTT with HAP1 depletes HAP1 from its normal functional site at the dynein/dynactin complex, negatively impacting axonal transport in HD (Gauthier et al., 2004).

These studies highlight the importance of HTT in normal synaptic function. It is no surprise then that various genetic therapies directed toward lowering mutant copy of HTT, which also reduced wild-type HTT level (Tabrizi et al., 2019), led to devastating consequences manifesting with motor and memory deficits (Kwon, 2021). It is interesting to note that both DNA damage and synaptic dysfunction are early signs of HD. However, there is no evidence that these two phenomena are linked or whether these processes are disrupted independent of one another in HD. One possibility is that synapses are disrupted because their energy demands are not met in HD. Most of the energy needed for synaptic function is supplied by mitochondria that localize to synapses and to the nodes of Ranvier in the motor neurons. There is plenty of evidence to suggest that mitochondrial function is disrupted in HD (Orr et al., 2008; Shirendeb et al., 2011; Siddiqui et al., 2012), and this could directly affect various synaptic processes. Second possibility is that in HD, there might a decrease in the interactions of HTT with its synaptic partners, much like we observed in DNA damage repair where mHTT can bind to the native DNA repair complex partners and decrease the efficacy of the complex to repair DNA damages (Gao et al., 2019). Finally, we know that inefficiency of DNA repair can disrupt transcription (Gao et al., 2019), which might affect the synaptic protein turnover leading to decreased synaptic function. However, to our knowledge, no study has yet demonstrated a direct link between DNA repair deficiency and synaptic dysfunction. Intriguingly, mutations that affect DNA repair have implications for many neurological diseases including ALS, AD, PD, and HD (Browne et al., 1997; Rolig and McKinnon, 2000; Bogdanov et al., 2001; Giuliano et al., 2003; Illuzzi et al., 2009; Bertoni et al., 2011; Madabhushi et al., 2014; Maynard et al., 2015; Askeland et al., 2018; Massey and Jones, 2018; Mitra et al., 2019; Shanbhag et al., 2019; Konopka et al., 2020). Thus, it would be interesting to investigate whether there is a direct link between synaptic demise and DNA repair and whether DNA repair is upstream of synaptic dysfunction in HD and related neurodegenerative diseases (Figure 1).

## Mutant Huntingtin Carrying Expanded Polyglutamine Sequences Impairs DNA Repair in Huntington’s Disease

It is often debated whether persistent accumulation of DNA damage that is consistently observed in HD brain is the cause or a consequence of HD pathophysiology. Emerging evidence indicates that mHTT interferes with the DNA repair process in the post-mitotic neurons, leading to the accumulation of DNA damage (Qi et al., 2007; Enokido et al., 2010; Tamura et al., 2011). The wide-spread occurrence of unresolved DNA damages/lesions in postmortem HD patient brains, and especially in the pre-symptomatic HD transgenic mouse brain early during the disease progression suggest that accumulation of DNA damage/lesions in HD could be a cause rather than a symptom of HD (Enokido et al., 2010; Tamura et al., 2011; Gao et al., 2019). However, these observations do not rule out the possibility that DNA damages accumulate independently due to other possible causes of neurodegeneration in HD. Indeed, both could contribute to the HD disease pathology but recent evidence from genome-wide association (GWAS) studies of HD have found many genes that are involved in DNA damage repair in neurons (e.g., FAN1, LIG1, MLH1, MSH3, PMS1, and PMS2) as strong modifiers of age at onset and disease severity (Genetic Modifiers of Huntington’s Disease [GeM-HD] Consortium, 2015; Maiuri et al., 2019; Tabrizi et al., 2020), suggesting that impaired or defective DNA repair mechanism is mechanistically linked to HD pathomechanism. Second, many recent studies support the idea that presence of DNA damage/lesions is one of the early pathological hallmarks in HD brain (Browne et al., 1997; Bogdanov et al., 2001; Giuliano et al., 2003; Illuzzi et al., 2009; Bertoni et al., 2011; Askeland et al., 2018; Massey and Jones, 2018), indicating that DNA damage occur early in the disease progression and persistence of these lesions might trigger a cascade of pro-degenerative pathways in HD. Third, the presence of substantially higher oxidized bases in genomic DNA, in cell and animal models of HD, and in human postmortem brains (Browne et al., 1997; Bogdanov et al., 2001; Giuliano et al., 2003; Illuzzi et al., 2009; Bertoni et al., 2011; Askeland et al., 2018; Massey and Jones, 2018) also indicate problems with DNA repair system(s) in HD. Importantly, accumulation of oxidized bases in DNA, with frequent DNA deletions in HD postmortem brains strongly support the idea (Horton et al., 1995; Browne et al., 1997; Polidori et al., 1999; Siddiqui et al., 2012). Fourth, transgenic expression of mHTT in mouse brain also induces DNA damage early in the disease progression (Bogdanov et al., 2001; Gao et al., 2019), suggesting that DNA repair system(s) that maintains neuronal genome integrity might be affected early in the disease progression. Finally, many studies have implicated HTT in double strand break (DSB) repair mechanism, and mHTT has been shown to interact with Ku70, an essential DSB repair protein and a regulatory component of the DNA-dependent protein kinase (DNA-PK) (Enokido et al., 2010). Interaction and sequestration of Ku70 by mHTT has been suggested to impair non-homologous end-joining (NHEJ)-mediated DNA DSB repair in HD neurons (Enokido et al., 2010). DSBs are the most lethal form of DNA lesions in postmitotic cells like neurons and unlike other cells, neurons do not use homologous recombination. Instead, NHEJ pathway is used in neurons to repair these potentially lethal lesions (Rass et al., 2007; McKinnon, 2009; Lieber, 2010). Importantly, stimulating DSB repair activity by overexpressing Ku70 in mouse or *Drosophila* model of HD can rescue neurodegeneration (Enokido et al., 2010; Tamura et al., 2011), suggesting that mHTT impairs NHEJ-mediated DSB repair in HD. These findings strongly support the idea that failure of the neuronal DNA repair mechanisms due to polyQ expansion in HTT could be an important early contributing factor to the downstream degenerative events commonly observed in HD. Why would the DNA repair process be so important and effect the neurons disproportionately? We know that most of the mature neurons in adult brains are postmitotic and these repair processes maybe more important in neurons than other cells that can undergo apoptosis in case of heavy damage to their DNA. Also, we know that neurons are metabolically highly active, even at resting state, generating excessive levels of potentially damaging reactive oxygen species (ROS), and these damaging free radicals have been demonstrated to induce various types of lesions in neuronal DNA, including DNA single-strand breaks, oxidative damages, base deletions, DSBs (Lindahl and Barnes, 2000). Therefore, if not efficiently resolved, these potentially lethal and damaging lesions can accumulate during routine neuronal activities and become highly toxic if left unresolved. Thus, while DNA damage might contribute to HD pathology, and that HTT may play a critical role in DNA repair processes in neurons, until recently, it was not clear how the native HTT protein participates in the DNA repair processes in normal neurons, and how polyQ expansions in mHTT disrupts this vital genome maintenance process in neurons.

### Huntingtin Facilitates DNA Repair by Stimulating Transcription-Coupled DNA Repair, and Mutant Huntingtin Impairs Transcription-Coupled DNA Repair in Huntington’s Disease

**FIGURE 2:**
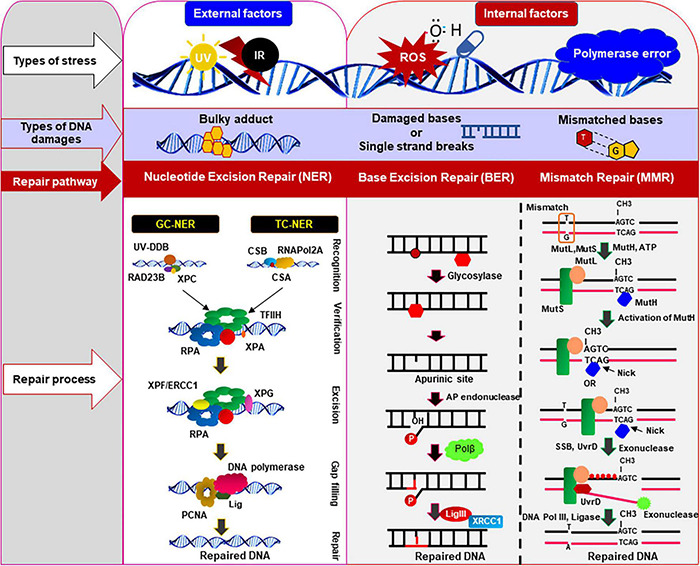
DNA damages and repair pathways in mammalian cells. Various types of DNA damages/lesions are routinely induced in genomic DNA by both internal genotoxic agents (e.g., reactive oxygen species: ROS, DNA polymerase error) as well as by external agents (e.g., ultra-violet radiation, infrared radiation, etc.). These potentially lethal damages are efficiently repaired by specialized DNA repair pathways, depending on types of DNA damages and cell types. This figure illustrates the common and simplified graphical presentation of the different types of DNA repair pathways involved to resolve various types of damages/lesions in mammalian cells in an error-free way. The set of repair proteins may vary between different pathways, and there might be some overlapping steps in different repair pathways. Nucleotide excision repair (NER), base excision repair (BER), and mismatch repair (MMR) pathways are the main repair pathways that protect genome from the internal or external genotoxic agents. These repair pathways play important role in repairing damages that routinely occur in genomic DNA of the post-mitotic neurons to maintain the sequence integrity and health of neurons. CSA/CSB, cockayne syndrome protein A/B; ERCC1, excision-repair cross-complementing 1; TFIIH, transcription factor IIH; RPA, replication protein A; GG-NER, global genomic nucleotide excision repair; TC-NER, transcription-coupled nucleotide excision repair; Lig, DNA ligase; RNAPol2A, RNA polymerase subunit 2A; XPA/XPC/XPF/XPG, proteins involved in different xeroderma pigmentosum (XP); PCNA, proliferating cell nuclear antigen; Polβ, DNA polymerase β; MLH, MutL protein homolog; MSH, MutS protein homolog; UV, ultraviolet; XRCC1, X-ray repair cross-complementing 1.

We know that the damaging, and potentially lethal lesions in neurons are generally faithfully and accurately resolved by a repertoire of DNA repair systems that strictly protect the genome integrity and health of mature neurons (Sancar et al., 2004; McKinnon, 2017). Oxidized DNA bases, abasic sites, single-strand breaks (SSBs), and DSBs are primarily repaired by the base-excision repair (BER) pathway in the central nervous system (Seeberg et al., 1995), and evidence to support the existence of nucleotide excision repair (NER), and mismatch repair (MMR) pathways in postmitotic neurons (Madabhushi et al., 2014; McKinnon, 2017). The plausible DNA damage repair pathways in mammalian cells are illustrated in Figure 2. However, since neurons are more vulnerable due to their high metabolic and transcriptional activities, and their postmitotic nature, there must be additional mechanisms that are important in protecting the neuronal genome of neurons. Indeed, emerging evidence including our own recent studies (Gao et al., 2015, 2019; Chakraborty et al., 2020) suggest that neurons have an additional and specialized repair mechanism in which the transcriptionally active genome is repaired more efficiently than the non-transcribing region to accurately maintain genomic integrity such that they do not produce defective or inactive proteins. How do long-lived cells like neurons accurately maintain their genome integrity despite constant exposure to endogenous genotoxic agents? Increasing evidence suggest that postmitotic cells like neurons have an additional layer of repair mechanism to protect their genome. It is hypothesized that the translocating RNA polymerase recruits specific DNA repair proteins to resolve the lesions during transcription elongation (Hanawalt, 1994; Hanawalt and Spivak, 2008). However, how in response to DNA damage, several DNA repair proteins are quickly assembled to accurately resolve the DNA lesions. However, how this process unfolds remains poorly understood, and needs further investigation. We have recently demonstrated that HTT plays a critical role in assembling a multi-protein transcription-coupled DNA single-strand break repair (TCR) complex, and that this novel multiprotein complex sense the DNA strand breaks/damages in the template DNA strand and resolves the DNA damages/lesions during transcription elongation (Gao et al., 2019). Our findings strongly suggest that the HTT-assembled macromolecular DNA repair-transcription complex provides an additional layer of protective mechanism to strictly maintain the sequence integrity of the protein-coding regions of neuronal genome. This specialized DNA repair system probably is an additional layer of protection evolved for postmitotic cells like neurons.

How does HTT help in the TCR process? HTT is a large (∼350 kDa) protein of 3,144 amino acids and many studies have shown that HTT is indispensable for neuronal development and survival (Duyao et al., 1995; Nasir et al., 1995; Zeitlin et al., 1995; Saudou and Humbert, 2016). While HTT is predominantly a cytosolic protein, it is also found to be present in the nucleus and mitochondria. HTT is enriched in the neurons, specifically in striatum, cerebral cortex, and cerebellum (Li et al., 1993; Sharp et al., 1995) and contains several copies of HEAT [huntingtin, elongation factor 3, protein phosphatase 2A (PP2A), and TOR1] motifs, which may adopt a tertiary structure that likely acts as a scaffold for the assembly of multiprotein complexes (Ochaba et al., 2014; Maiuri et al., 2017). In the nucleus, HTT also interacts with RNA polymerase and various transcription factors (Giuliano et al., 2003; Illuzzi et al., 2009; Bertoni et al., 2011; Ross and Tabrizi, 2011; Lu et al., 2014; Valor, 2015; Jimenez-Sanchez et al., 2017). Previous studies have also implicated HTT in DNA repair due to its interaction with ku70, an essential DNA repair protein (Enokido et al., 2010). Importantly, restoring DNA repair by overexpressing Ku70 in mouse or *Drosophila* model of HD was found to rescue neurodegeneration (Enokido et al., 2010; Tamura et al., 2011), suggesting that mHTT impairs DNA repair in HD and that DNA damage may be somehow linked to the demise of the neurons.

Our recent studies have shown that HTT organizes a multi-protein TCR complex with RNA polymerase, transcription factors and cofactors (e.g., TBP, CBP, TAF4, etc.), ataxin-3, a deubiquitinating enzyme and DNA repair enzymes that include DNA ligase 3 and polynucleotide kinase 3′-phosphatase (PNKP) (Gao et al., 2019). Interestingly, role of PNKP was implicated in DNA repair deficiency in polyQ expansion disease(s) pathology previously (Barclay et al., 2014). Our data indicate that the multiprotein TCR complex assembled by HTT stimulates DNA strand break repair in neurons by stimulating the end-processing activity of the DNA strand break repair enzyme PNKP (Gao et al., 2019). By contrast, our study has shown that the presence of mHTT in the TCR complex dramatically decreases the activity of PNKP, leading to persistent accumulation of DNA damages in HD (Gao et al., 2019). mHTT-mediated inactivation of the TCR complex in neurons results in preferential accumulation of DNA strand-breaks/damages within the transcriptionally active genome and the number of damages is significantly lower in the genomic regions that are not transcriptionally active in the neurons (Hanawalt, 1994; Hanawalt and Spivak, 2008; Chakraborty et al., 2016, 2020; Gao et al., 2019).

Moreover, the chromatin immunoprecipitation (ChIP) experiments showed that HTT and associated TCR complex components associate with the transcriptionally active genome compared with the inactive genome and the former accumulates more strand breaks/damages in the HD or SCA3 brains (Gao et al., 2019; Chakraborty et al., 2020). ChIP analysis revealed a significantly higher occupancy of HTT on the actively transcribing genome in the brain [e.g., neuronal differentiation factor 1 and 2 (Neurod1 and Neurod2), neurogenic basic-helix-loop-helix protein neurogenin 1 (Neurog1), tubulin beta 3 class III (Tubb3), neuron-specific enolase 2 (Eno2γ), and DNA polymerase beta (Pol b)] over genes that are not transcribed in the brain but actively transcribed in skeletal or cardiac muscle [e.g., myogenic differentiation factor 1 (Myod1); myogenic factor 4; myogenin (Myog); and myosin heavy chain 2, 4, 6, or 7 (Myh2, Myh4, Myh6, or Myh7); Gao et al., 2019]. Increased association between HTT with the transcriptionally active genome and mHTT-mediated abrogation of TCR complex activity suggest that HTT-assembled TCR complex predominantly repairs the DNA lesions during transcription elongation, but polyQ expansion in HTT might impair the TCR activity, resulting in DNA damage accumulation predominantly within the actively transcribing regions of genome in HD. Indeed, analysis of genomic DNA revealed 60–70% lower PCR-amplification of actively transcribing genes in asymptomatic (7 weeks old) transgenic zQ175 mouse brains compared to age-matched controls (Gao et al., 2019). In contrast, the amplification efficacy for non-transcribing genes in the zQ175 brains was only marginally (10–15%) reduced (Gao et al., 2019), indicating significantly less DNA strand break accumulation. These data suggest that HTT-TCR complex repairs the strand breaks that routinely accumulate during transcription, and that this function is impaired by polyQ expansion in HTT, resulting in persistent damage accumulation predominantly affecting the actively transcribing genes in neurons.

Persistence of DNA lesions/damages within the actively transcribing genes or promoter regions may impede transcription of a variety of neuronal genes, including the genes regulating synaptic function, vesicular transport, and calcium homeostasis, impacting overall neuronal health and function, contributing to neurotoxicity at the early stages in HD disease progression. It is tempting to speculate that inefficient TCR is an important contributor to the accumulation of DNA lesions, and transcriptional dysregulation, hallmark features of the HD (Browne et al., 1997; Ayala-Peña, 2013). Therefore, based on our data (Gao et al., 2019), we hypothesize that the combination of reduced HTT levels, and the presence of mHTT in the TCR complex causes DNA repair deficiency resulting in persistent accumulation of DNA damages in the neuronal genome, triggering early neurotoxicity and neurodegeneration in HD. Importantly, this raises the possibility that HTT may act as an interchangeable scaffold to organize components for different DNA repair processes as previously suggested (Qi et al., 2007). Also, we refer the readers to recent excellent reviews on the topic of various types of neuron-specific DNA repair mechanism and neurodegeneration (Jonson et al., 2013; Jones et al., 2017; Ross and Truant, 2017). Taken together, these studies provide compelling evidence to indicate that HTT-assembled TCR complex helps to resolve the DNA lesions during active transcription, and this mechanism might provide an additional protective mechanism to preserve the sequence integrity of the protein-coding regions of the neuronal genome.

**FIGURE 3:**
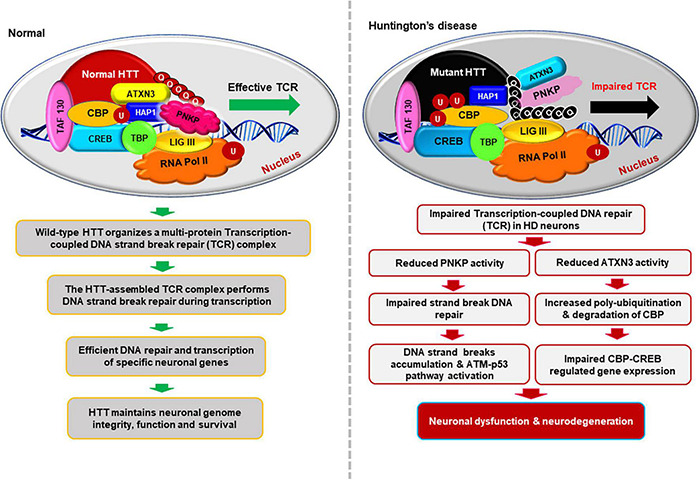
Schematics illustrating how HTT helps to maintain neuronal genome integrity and how mHTT synchronously impairs DNA repair and transcription in HD. This hypothesized multi-protein transcription-coupled DNA repair (TCR) complex assembled by HTT senses DNA strand breaks/lesions during transcription elongation and orchestrates their faithful repair to maintain the sequence integrity of neuronal genome. HTT thus plays a pivotal role in maintaining neuronal health, function, and survival. This novel complex thus provides an additional layer of protective mechanism to strictly maintain the sequence integrity of the transcriptionally active region of genome to protect the protein-coding regions of neuronal genome. By contrast, the presence of mHTT in the TCR complex impairs the activity of the complex, resulting in persistent accumulation of DNA lesions/damages in neuronal genome. Persistence of DNA strand breaks/lesions in genome can interfere with the translocation of transcribing RNA polymerase over the template DNA, impeding adequate transcription of many neuronal genes in HD. Inadequate expression of neuronal genes, may adversely impact neuronal health, neurotransmission, calcium homeostasis, and synaptic function, causing early neurotoxicity in HD. Additionally, persistence of unresolved DNA damage in neuronal genome may also result in chronic activation of the DNA damage-response (DDR) ATM-p53-dependent pro-apoptotic signaling pathways facilitating premature demise of neurons in HD. Moreover, mHTT also can inactivate ATXN3’s deubiquitinating activity, facilitating poly-ubiquitination and subsequent degradation of cyclic AMP-response element-binding protein (CBP), impairing CBP-CREB-regulated gene transcription, further amplifying the pro-degenerative output in the HD neurons.

Based on the data and what is already known about DNA damage repair and HTT, we propose a model in which RNA polymerase-mediated transcription pausing at DNA damage sites leads to phosphorylation-dependent activation of the DDR kinase ATM and DNA-PK, two of the important serine-threonine kinase that are phosphorylated and activated in response to DNA damage (Kurz and Lees-Miller, 2004; Guo et al., 2010; Yue et al., 2020). Phosphorylation of ATM and/or DNA-PK enhances phosphorylation-dependent recruitment of PNKP (Segal-Raz et al., 2011; Zolner et al., 2011) at the damaged sites, facilitating DNA damage repair. The presence of DNA lesions thus converts the translocating HTT-POLR2A transcription complex into an active TCR complex assisted by HTT. After repair, specific repair factor(s) are dephosphorylated by protein phosphatases, and the transcription complex restarts the paused transcription (Figure 3). When TCR activity is disrupted in neurodegenerative diseases like HD, the complex stalls at DNA lesions, failing or delaying the initiation of repair, compromising the genome integrity and transcription resulting in neuronal dysfunction, and premature death of neurons.

### Mutant Huntingtin Impairs DNA Repair by Interfering With the Expression and/or Activity of the High Mobility Group Proteins in Neurons

A large number of studies have demonstrated that both wild-type HTT as well as mHTT carrying extended polyQ are present within the nuclei, and interact with several nuclear transcription factors and cofactors (Huang et al., 1998; McCampbell et al., 2000; Shimohata et al., 2000b; Steffan et al., 2000; Nucifora et al., 2001; Suhr et al., 2001; Dunah et al., 2002; Bae et al., 2005; Zhai et al., 2005; Cha, 2007; Benn et al., 2008; Ross and Tabrizi, 2011; Valor, 2015; Jimenez-Sanchez et al., 2017). Interactions of mHTT with the nuclear transcription factors have been implicated in extensive transcription dysregulation observed in HD (Ross and Tabrizi, 2011; Valor, 2015; Jimenez-Sanchez et al., 2017). In addition to these transcription factors, high mobility group proteins B1 and B2 (HMGB1/2) were also found to interact with the mutant form of HTT and ataxin-1, another polyQ-containing protein (Qi et al., 2007). HMGB proteins are evolutionarily conserved non-histone chromatin-associated proteins that play key roles in maintaining nuclear homeostasis. However, the function of HMGBs in the nuclei of brain cells is poorly understood. Interestingly, HMGB proteins are significantly reduced in the nuclear region outside of inclusion bodies in the affected neurons in HD and spinocerebellar ataxia type 1 (SCA1) (Qi et al., 2007). Furthermore, expression of HMGB proteins was found to significantly ameliorate polyQ-induced pathology in primary neurons as well as in *Drosophila* models of polyQ diseases (Qi et al., 2007), suggesting that HMGB proteins may be critical regulators of polyQ-mediated neurotoxicity. Furthermore, it was also demonstrated that reduction of nuclear HMGB protein level in the nucleus strongly correlated with DSB-mediated neuronal damage in HD (Qi et al., 2007). Moreover, HMGB1 protein was found to localize to neuronal nuclei, and its protein levels changed in various brain regions in an age-dependent manner (Enokido et al., 2008). Intriguingly, reduced expression of HMGB proteins correlated with the increased incidence of DSBs in neurons as compared with the astrocytes. These findings indicate that HMGB expression levels during aging might be an important indicator for DNA DSBs in neurons (Figure 1).

Finally, while there is overwhelming evidence that suggests that DNA repair mechanism(s) fail in HD and many other microsatellite repeat expansion related neurodegenerative disorders, there is some evidence in R6/1 mouse model of HD where it was shown that DNA repair system *per se* is not affected (Kovtun et al., 2007). These results while intriguing, need further evaluation in other models of HD and other related diseases.

### Mutant Huntingtin Transcripts Encoding Extended CAG RNA Sequences Can Induce DNA Damage to Trigger Neurotoxicity in Huntington’s Disease

Emerging evidence suggest that the mHTT transcripts carrying the expanded CAG RNA sequences can also contribute to genotoxicity in HD (Bañez-Coronel et al., 2012; Martí, 2016; Peng et al., 2021). A recent study by Peng et al. (2021) has raised the possibility that small RNA molecules encoding CAG sequences, generated from the cleavage of the mHTT transcripts, can contribute to DNA damage in HD. In this study, the expression of the *nudix hydrolase 16* (*NUDT16*) gene was found to be downregulated in CAG RNA expressing cells and in mouse model of HD (Peng et al., 2021). Amongst its many functions, NUDT16 is an RNA de-capping enzyme that catalyzes the cleavage of the cap structure of small nucleolar RNAs (snoRNAs) and mRNAs. It also has a diphosphatase activity that removes m7G and/or m227G caps from U8 snoRNA and leaves a 5′monophosphate on the RNA (Iyama et al., 2010; Trésaugues et al., 2015). Importantly, Peng et al. (2021) have shown that loss of NUDT16 function in cell and animal model of HD results in misincorporation of damaging nucleotides into DNAs leading to DNA damage accumulation. These findings suggest that small CAG RNAs can hybridize with CUG-containing *NUDT16* transcripts and form a CAG-CUG RNA heteroduplex, resulting in silencing of *NUDT16* gene, which may lead to the DNA damage accumulation and neuronal apoptosis in HD (Peng et al., 2021).

## Mutant Huntingtin-Induced DNA Damage Activates the DNA Damage-Response Signaling to Trigger Neurotoxicity in Huntington’s Disease

A recent study highlighting the importance of DNA damage in neurodegenerative disorders has shown that either genetic or pharmacological ablation of DDR kinase ATM activity significantly ameliorates neurotoxicity in HD animal models (Lu et al., 2014). This report strongly supports the emerging view that persistent accumulation of unresolved DNA damage in neuronal genome and the subsequent chronic activation of the DDR ATM signaling pathway probably is one of the major contributing factors in HD pathogenic mechanism(s). Our recent publication also supports this idea because the presence of mHTT or mutant ataxin-3 in the transcription-linked DNA repair complex dramatically impairs its DNA repair activities, resulting in DNA damage/strand break accumulation and chronic activation of the DDR pathway in HD (Gao et al., 2019) as well as in SCA3 (Gao et al., 2015) respectively. Furthermore, our study suggests that HTT may help stimulate TCR complex activity and DNA repair, while mHTT reduces DNA repair activity thereby, impairing DNA damage repair and enhancing the DNA strand breaks in genome.

Persistent and cumulative accumulation of DNA damage/strand breaks can result in chronic activation of the DDR serine-threonine kinase ATM as well as DNA-PK, which in turn phosphorylate the tumor suppressor protein p53 (Kurz and Lees-Miller, 2004; Guo et al., 2010). The transcription factor p53 is the primary target of DDR pathway, and many of the functions of ATM/DNA-PK are p53-dependent and activated p53 regulates a wide variety of cellular processes such as transcription, cell-cycle regulation, DDR and DNA repair, and cell death (Miller et al., 2000; Kurz and Lees-Miller, 2004; Culmsee and Mattson, 2005; Chang et al., 2012). Aberrant activation of p53 pathway has also been reported in several other polyQ-expansion-associated hereditary ataxias including HD (Bae et al., 2005; Wang et al., 2010; Chou et al., 2011). The activated p53 (phosphorylated p53) can transactivate expression of many proapoptotic genes such as BAX, Bcl2L2 (encoding BIM), PMA1P1 (encoding NOXA), BBC3 (encoding PUMA), triggering apoptotic pathways (Oda et al., 2000; Nakano and Vousden, 2001; Shibue et al., 2003; Chipuk et al., 2004, 2005; Cregan et al., 2004; Kuwana et al., 2005; Gao et al., 2015). Persistence of unrepaired DNA damage in genome thus can facilitate p53-depndent activation of pro-degenerative pathways in HD as described earlier (Bae et al., 2005). In response to DNA strand breaks, ATM directly phosphorylates p53, which in turn activates transcription of many pro-apoptotic genes (Oda et al., 2000; Chipuk et al., 2004). Expression of mHTT has been shown to increase p53 target gene expression, whereas deleting p53 in the HD transgenic mouse brain rescues the behavioral abnormalities (Bae et al., 2005) highlighting the fact that mHTT-mediated chronic activation of DDR-p53 pathway contributes to neurotoxicity in HD. These data indicate that HTT is important in DNA strand break repair, whereas mHTT impairs DNA repair activity, resulting in DNA strand break/damage accumulation, and cumulative accumulation of DNA damage results in chronic activation of ATM-dependent p53 signaling pathway to triggers neurotoxicity in HD.

**FIGURE 4:**
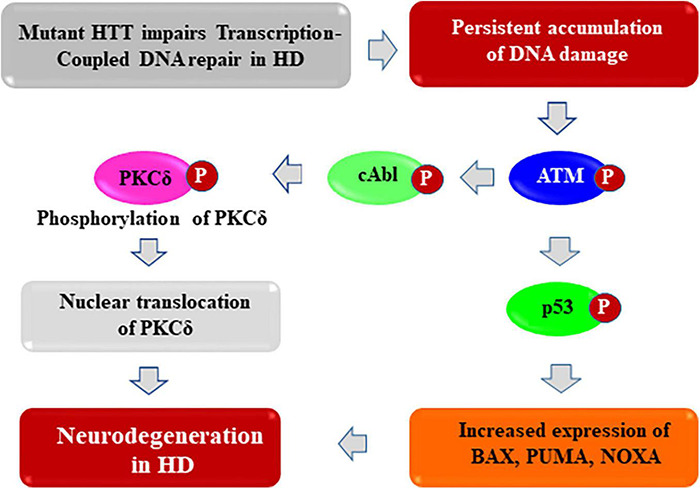
Proposed mechanism by which mHTT activates pro-apoptotic signaling to trigger neurotoxicity in HD. The huntingtin (HTT) protein probably acts as a structural scaffold to assemble a novel macromolecular transcription-coupled DNA repair (TCR) complex with RNA polymerase large subunit, various transcription factors and cofactors, and DNA repair enzymes including DNA ligase 3 (LIG 3) and DNA strand break repair enzyme PNKP (polynucleotide kinase 3′-phosphatase). This novel multi-protein complex senses the DNA strand breaks/lesions during transcription elongation and orchestrates their faithful repair to strictly maintain genome integrity. By contrast, the presence of mHTT in the TCR complex impairs its activity resulting damage accumulation in neuronal genome. This results in enhanced phosphorylation of DNA damage-response serine-threonine kinase ATM, and DNA-dependent protein kinase (DNA-PK), leading to chronic activation of ATM and/or DNA-PK-dependent p53 and c-Abl kinase signaling cascade in HD. This trigger pro-apoptotic signaling pathways by enhancing expression of various p53 target genes such as BAX, PUMA, and NOXA in HD. In parallel, activated ATM/DNA-PK can also phosphorylate the c-Abl kinase, which in turn phosphorylate PKCδ, facilitating nuclear translocation of PKCδ. Nuclear translocation of PKCδ may further amplify the pro-apoptotic signal in HD, ultimately leading to neuronal degeneration and development of complex neurological phenotypes in HD.

Furthermore, persistence of DNA damage can also trigger pro-apoptotic signaling in another ATM and/or DNA-PK-dependent but p53-independent pathway in HD. There is ample evidence suggesting that persistent accumulation of DNA damages and subsequent phosphorylation-dependent chronic activation of the DDR kinase ATM and/or DNA-PK can also result in the phosphorylation of another downstream tyrosine kinase c-Abl (encoded by the mammalian homolog of the v-Abl oncogene from the Abelson murine leukemia virus) (Baskaran et al., 1997; Kharbanda et al., 1997; Shafman et al., 1997). Experimental evidence suggests that the activated c-Abl kinase (phosphorylated form of c-Abl) can constitutively associates with the protein kinase C delta (PKCδ), resulting in phosphorylation and subsequent nuclear translocation of PKCδ (Yuan et al., 1998; Yoshida, 2008; Adwan et al., 2011). Several studies have shown that cytosolic retention of PKCδ is required for cell survival and function while its phosphorylation and subsequent nuclear translocation can activate the apoptotic pathways (Basu et al., 2001; Yoshida, 2008; Adwan et al., 2011). However, it remains to be tested whether chronic activation of the DDR pathways and aberrant activation of the c-Abl→PKCδ pathway contributes to degeneration of neurons in HD. The presence of unrepaired DNA damages in neuronal genome and subsequent chronic activation of the DNA damage sensor ATM and/or DNA-PK kinases thus can concomitantly activate several pro-degenerative signaling pathways in neurons, and presence of chronic but low-grade DDR pathways in neurons can lead to neuronal dysfunction, and finally their premature demise in HD (Figure 4).

In response to DNA damage, several DDR kinases e.g., ATM, ATR, DNA-PK, p53, chk1 and 2 (checkpoint kinase 1 and 2), etc. are phosphorylated, and the phosphorylated form of these effector DDR kinases coordinately regulate DNA repair, neuronal survival, and their fate (Kurz and Lees-Miller, 2004; Guo et al., 2010). The important question that needs vigorous investigation is whether blocking ATM and/or DNA-PK with small molecule inhibitors can block neurodegeneration in HD. Therefore, significant efforts are being made to develop therapeutic approaches to block ATM or DNA-PK kinases (Lu et al., 2014). It is important to note that the DDR kinase also play important role in mediating DNA repair processes in the neurons by triggering phosphorylation-dependent activation of DNA damage repair proteins such as BRCA1, p53, PNKP, Ku70, ligase IV, etc. Therefore, one must consider the possibility that therapeutic approaches for HD aiming to block the DDR kinases with small molecule inhibitors may lead to impaired DNA repair in neurons, resulting in adverse consequences. Therefore, understanding the detailed molecular mechanism of DNA repair in neurons, and how polyQ expansion in HTT causes neurotoxicity affects these processes will provide more rational and targeted therapeutic strategies.

## How Does Impaired TCR Predominantly Affect Striatal Neurons in Huntington’s Disease?

An important unanswered question is how mHTT impacts specific neuronal populations in HD despite its ubiquitous expression in the brain cells. Why do different neurons and/or brain regions show variable sensitivity to genotoxic insults? Neither the transcriptional signatures nor the activity patterns of any two classes of neurons are likely to be identical; therefore, the regions of robust and high-fidelity DNA repair, as well as those that are poorly maintained, likely vary from neuron to neuron. Although our findings suggest that the efficiency of TCR predominantly impacts the cortex and striatum with little effect on the cerebellar DNA, in HD, they do not explain why mHTT predominantly impairs TCR in specific brain regions in HD. How might inactivation of a potentially key DNA repair mechanism like TCR differentially affect various brain regions in HD? One possibility is that several factors specifically regulate the activity of this complex in various brain regions, particularly under genotoxic stress conditions. Inactivation of these region-specific regulators could potentially be impacted differentially causing the difference in the levels of DNA damage in different brain regions. Alternatively, specific brain regions might be differentially vulnerable to impaired TCR complex function. Therefore, complete, and detailed elucidation of the composition of this unique transcription-linked DNA repair complex, particularly in different brain regions and understanding how its activity is regulated by these factors may clarify the selective vulnerabilities of neurons in the HD brain.

While it is often argued that ineffective DNA repair and/or cumulative accumulation of unresolved DNA lesion/damages in neuronal genome are the consequences, and not the causes of neuronal dysfunction, toxicity and degeneration, we diverge and hypothesize that in fact, impaired or defective DNA repair might be an early pathology in not only in HD but also in several other terminal neurodegenerative diseases like ALS (Konopka and Atkin, 2018; Mitra et al., 2019; Konopka et al., 2020), AD (Shackelford, 2006; Shanbhag et al., 2019), PD (Milanese et al., 2018; Schaser et al., 2019; Martín-Jiménez et al., 2020; Gonzalez-Hunt and Sanders, 2021), SCA7 (Niss et al., 2021), and SCA3 (Chatterjee et al., 2015; Gao et al., 2015; Chakraborty et al., 2020). It is possible that once DNA damage occurs due to faulty or impaired repair system, the presence of highly damaging and potentially lethal lesions may become irreversible in adult post-mitotic neurons, and no interventions will rescue the damages or phenotype after that point. However, our data argue against this point. Our goal must therefore be to prevent neurons from reaching that critical tipping point and identify therapeutic targets within this window of opportunity to stop or reverse the degeneration for diseases like HD.

## Concluding Remarks

Studies showing that brain-specific knockout of *HTT* in postnatal mouse brain triggers neurodegeneration (O’Kusky et al., 1999; Dragatsis et al., 2000), suggesting that HTT plays important roles in neuronal development and survival. On the other hand, immortalized cell lines or primary neurons overexpressing HTT are resistant to degeneration following exposure to degenerative stimuli including expression of mHTT, and transgenic mice overexpressing HTT show tremendous resistance to degeneration when exposed to excitotoxic and ischemic injury (Rigamonti et al., 2000; Ho et al., 2001; Zhang et al., 2003, 2006; Leavitt et al., 2006). Although these reports suggest that increased HTT levels dramatically improve neuronal resistance to degeneration, the precise mechanism by which this protein provides neuroprotection remains poorly understood. Emerging evidence, including our studies, suggest that HTT plays a critical role in DNA strand break repair possibly by assembling the macromolecular TCR complex. Based on our recent data we hypothesize that this complex “senses” DNA damage/lesions in the template DNA and orchestrates their repair during transcription to maintain sequence integrity of neuronal genome. On the other hand, mHTT-mediated loss of DNA repair and deubiquitinating activities might be critical proximal events that impair DNA repair as well as transcription in HD. This mechanism would provide a mechanistic link between transcriptional dysregulation, cumulative DNA damage accumulations and inappropriate and chronic activation of the ATM/DNA-PK-dependent pro-apoptotic pathways, which trigger early neurotoxicity in HD. While the final biological output triggered by impaired TCR and persistence of unrepaired DNA damages in HD remains to be understood, our study points to a possible TCR mechanism that may be disrupted by mHTT in HD (Gao et al., 2019). The molecular strategies that interfere with the interaction of mHTT with the TCR complex might help slow neurotoxicity and the functional decline in HD. Alternatively, molecular approaches to stimulate PNKP activity for efficacious DNA repair may be another way to combat transcriptional dysregulation in HD. Collectively, the various studies on DNA repair deficiency in HD (Qi et al., 2007; Enokido et al., 2010; Tamura et al., 2011) and our recent findings (Gao et al., 2019) may help clarify how mHTT compromises genome integrity and neuronal function in HD.

## Author Contributions

SP, RG, NZ, and KB contributed to the experiments described in this review article. PS and YW wrote and edited the manuscript. All authors read and contributed to the editing of the manuscript and approved the submission of all versions of the review article.

## Conflict of Interest

The authors declare that the research was conducted in the absence of any commercial or financial relationships that could be construed as a potential conflict of interest.

## Publisher’s Note

All claims expressed in this article are solely those of the authors and do not necessarily represent those of their affiliated organizations, or those of the publisher, the editors and the reviewers. Any product that may be evaluated in this article, or claim that may be made by its manufacturer, is not guaranteed or endorsed by the publisher.
